# Sputum interleukin (IL)-13 as a biomarker for the evaluation of asthma control

**DOI:** 10.1186/2045-7022-5-S2-O9

**Published:** 2015-03-23

**Authors:** Zoi Tsilogianni, Petros Bakakos, Andriana I Papaioannou, Anastasia Papaporfyriou, Georgios Hillas, Spyros Papiris, Nikolaos Koulouris, Stelios Loukides, Konstantinos Kostikas

**Affiliations:** 1 Sotiria, General Hospital of Chest Diseases, 1st Respiratory University Medicine Department, Athens, Greece; 2 Attikon General University Hospital, 2nd Respiratory University Medicine Department, Athens, Greece

## Background

The management of patients with severe refractory asthma (SRA) is a difficult task that may benefit from the use of non-invasive assessment by biomarkers. Interleukin (IL)-13 is a cytokine with a central role in the TH2 response. The object of this study was the assessment of IL-13 as a biomarker of asthma control in patients with SRA.

## Method

We recruited 170 patients (47 with SRA) from two University asthma clinics. Patients were submitted to pulmonary function testing, measurement of fractional exhaled nitric oxide (FeNO), and sputum induction with calculation of inflammatory cells and measurement of IL-13 in the supernatant. Asthma control test (ACT) was used for evaluation of asthma control (ACT values >19 identified well-controlled asthma). Severe refractory asthma was defined by the ATS criteria.

## Results

IL-13 in sputum supernatant was increased in patients with SRA compared to patients with mild-to-moderate asthma [median (IQR): 156 (80-245) pg/mL vs. 78 (66-103) pg/mL, p<0.001], as well as in patients with persistent airflow obstruction [99 (71-199) pg/mL vs. 80 (66-105) pg/mL, p=0.0081]. IL-13 presented good diagnostic accuracy for the detection of not-well-controlled asthma in the whole group [specificity 94.2%, sensitivity 80.6% for a cut-off point of ≤ 156 pg/mL; area under the curve (AUC) 0.920]. The diagnostic accuracy of IL-13 was superior to FeNO or sputum eosinophils in patients with SRA (AUC 0.981 vs. 0.688 vs. 0.645 respectively; p<0.001).

## Conclusion

IL-13 in induced sputum may represent a useful index of disease control in patients with SRA. The better diagnostic accuracy of IL-13, compared to FeNO and sputum eosinophils, indicates a central role of this mediator in SRA.

